# Early Response of the Orthopaedic Trauma Community to the COVID-19 Pandemic

**DOI:** 10.7759/cureus.11883

**Published:** 2020-12-03

**Authors:** Colin K Cantrell, Bennet A Butler, Michael Stover

**Affiliations:** 1 Orthopaedics, Northwestern University Feinberg School of Medicine, Chicago, USA

**Keywords:** covid-19, corona pandemic, orthopedic trauma

## Abstract

Background

With the rise of the COVID-19 pandemic, medical systems and providers have been forced to undertake substantial changes to staffing structure, hospital policy, and surgical indications to remain able to care for patients and protect the providers. Here, we present a survey of orthopaedic trauma fellowship directors to assess when and what changes these individual units have made in order to cope with this global pandemic.

Methods

The survey was distributed via email to all 62 programs listed in the Orthopaedic Trauma Association web site.

Results

Twenty four responses were received. The majority of programs implemented changes between March 1^st^ and 14^th^, with nearly all splitting teams into individual units, incorporating virtual sign out, and reducing the number of available, i.e. exposed, staff, fellows, and residents at any particular time.

Conclusions

These changes have been implemented in order to proactively maintain the functionality of these vital teams to patient care with no definite end point to this pandemic in sight. We hope this study provides other trauma centers and orthopaedic practices insight into possible precautions that can be taken in response to the COVID-19 pandemic.

## Introduction

Since its identification and spread throughout China in late 2019, COVID-19 has transformed into a global pandemic of catastrophic proportions [1,2]. Medical systems across the globe have seen a large influx of COVID-19 patients [3]. These patients threaten to overwhelm the existing hospital space and staff in many areas. This problem is compounded by a relatively high rate of infection among healthcare professionals in severely affected regions [4]. In response to this crisis, many hospitals cancelled some or all elective surgeries to free up hospital space and decrease potential exposure events in the hospital [5]. 

Emergent and urgent surgical procedures, however, continue to be a priority. For this reason, orthopaedic trauma surgery teams have remained active [6]. Given the infectivity of COVID-19, many of these teams have been required to alter their structure, both to protect their team members from infection and to ensure that their team is not incapacitated to such an extent that they cannot provide care to their patients.

Here we use survey data to assess the early response of orthopaedic trauma teams to the COVID-19 pandemic. This data may be useful for informing the decisions of orthopaedic trauma team leaders and/or orthopaedic department leaders responding to this and potentially future pandemics.

## Materials and methods

This study was exempt from Institutional Review Board approval as all information was gathered without identifiable information in an anonymous survey. A list of thirteen questions, comprised by the authors, detailing possible responses and changes due to the COVID-19 pandemic on the orthopaedic trauma community was organized into a survey. Data deemed important by the authors included changes implemented by the hospital and orthopaedic trauma team and the timelines of those implemented changes. We also wanted to assess the respondents’ satisfaction to those changes, as well as perceived future directions. Understanding which, if any providers, tested positive was also crucial in understanding who may be at the most risk of exposure. A full list of survey questions with answer choices is available in Table 1.

**Table 1:** OTA Fellowship Director’s Survey

OTA Fellowship Directors’ Survey Questions and Answer Choices
1. Please select the Region in which you practice:
a. Northeast (Maine, New Hampshire, Vermont, Massachusetts, Rhode Island, Connecticut, New York, New Jersey, Pennsylvania)
b. Midwest (Ohio, Michigan, Indiana, Wisconsin, Illinois, Minnesota, Iowa, Missouri, North Dakota, South Dakota, Nebraska, Kansas)
c. South (Delaware, Maryland, Virginia, West Virginia, Kentucky, North Carolina, South Carolina, Tennessee, Georgia, Florida, Alabama, Mississippi, Arkansas, Louisiana, Texas, Oklahoma)
d. West (Montana, Idaho, Wyoming, Colorado, New Mexico, Arizona, Utah, Nevada, California, Oregon, Washington, Alaska, Hawaii)
2. When did your team start making taking precautions against COVID:
a. Prior to March 1^st^
b. March 1-14
c. March 15-31
d. April 1^st^ to Present
e. No changes made
3. What precautions has your center taken up to this point:
a. Split service into teams
b. Virtual (Phone, Video, etc) sign-out
c. Standard PPE for all patients
d. N95 masks in the OR
e. Decrease in available residents/fellows at a particular time
f. Other: please specify
4. If your service is split into teams, how is the rotation among teams structured?
a. Every other day
b. # of consecutive days: specify how many
c. Other: please specify
d. Not split into teams
5. Have/when elective cases been postponed/cancelled?
a. Yes (please specify when)
b. No
6. How many dedicated orthopaedic trauma operating rooms were available pre-COVID?
a. 0
b. 1
c. 2
d. 3
e. 4
f. 5+
7. How many dedicated orthopaedic trauma ORs are available currently?
a. 0
b. 1
c. 2
d. 3
e. 4
f. 5+
8. Has your case volume:
a. Increased
b. Decreased
c. Relatively Unchanged
9. What types of cases are still occurring at your institution:
a. Compartment Syndrome
b. Septic joints
c. Open Fractures
d. Closed Fractures
e. Nonunion/Malunion
f. Tendon/ligament repair
g. Irreducible dislocations
h. Other: please specify
10. With respect to fractures, are there any fractures that you usually treat operatively that are being elected to treat non-operatively or referred to different institutions:
a. Yes, please specify:
b. No, we have not changed our indications for operative fracture treatment
11. Have any of your staff tested positive for COVID (If Yes, When)?
a, Attending staff
b. Fellows
c. Residents
d. Ancillary staff
e. None
12. Are you satisfied with the precautions you have taken thus far?
a. Yes
b. No, please specify
13. Are there any additional precautions that you have considered, but not yet implemented?
a. Yes, please specify
b. No

The survey was constructed using SurveyMonkey (San Mateo, California, USA).

Once the survey preparation was complete, a list of Fellowship Directors from the Orthopaedic Trauma Association approved Fellowships was obtained from the Orthopaedic Trauma Association (OTA) web site [7]. Regions of practice in the survey were defined by data published by the United States Census Bureau [8]. 

The survey was distributed via email by one author. Fellowship director email addresses were used when available in the OTA fellowship directory. If a fellowship director's email address was not available for a program, the program coordinator's email address was then substituted.

Results were collected using SurveyMonkey and exported to Microsoft Excel (Redmond, Washington, USA) for statistical analysis.

## Results

Survey Respondents

Sixty-two accredited orthopaedic trauma programs were listed on the OTA web site. Fifty-seven (92%) programs contained email addresses for the program director (PD). For the five programs which did not list a PD email address, the program coordinator email address was substituted. Twenty four (39%) responses were received. The most responses were received by programs classified in the Midwest region (9), followed by South (8), West (6), and lastly Northeast (1).

Changes to Orthopaedic Trauma Team Structure

The majority of responding program directors (14; 58%) reported implementation of changes to their team structure in response to the COVID-19 epidemic beginning between March 1st and 14th, followed closely by nine (38%) programs reporting employing changes between March 15th and 31st. Only one program reported taking precautions prior to March 1st. The two most common changes implemented were splitting teams into self-contained units to decrease mass exposure events among team members and transition to a virtual sign out, each of which was reported by 88% of respondents. With regards to splitting teams into self-contained units, fourteen programs reported rotating these units on and off service for some number of consecutive days in a row, while five programs reported rotating these units on alternate days. Thirteen (54%) programs reported utilizing standard personal protective equipment (PPE), while only 10 (42%) programs reported utilizing N-95 masks in the operating room. Two programs reported separating clinic and surgical teams, one specifying that residents no longer participated in clinic duties. Further data regarding changes implemented to team structure are detailed in Table 2.

**Table 2:** Orthopaedic Trauma Implemented COVID-19 Precautions PPE: Personal protective equipment

Precaution	Number (Percentage)
Split service into teams	21 (88%)
Virtual (Phone, Video, etc) Sign-out	21 (88%)
Decrease in available residents/fellow at a particular time	20 (83%)
Standard PPE for all patients	13 (54%)
N-95 Masks in operating room	10 (42%)
Other	4 (17%)

Changes in Operative Volumes and Indications

Eleven program directors reported no decrease in dedicated orthopaedic trauma operating rooms, while thirteen reported a decrease in available operating rooms. Twenty-four (79%) respondents reported decreased acute trauma case volumes, while only five (21%) reported no change.

All respondents reported that elective cases have been postponed or cancelled at their institutions (5). Only four programs (17%) continue to perform nonurgent malunion and nonunion cases. In contrast, all programs continue to perform surgery as usual for open fractures, septic joints, and compartment syndrome (Table 3).

**Table 3:** Types of Operative Orthopaedic Trauma Cases Still Being Performed

Case Type	Number	Percentage
Compartment Syndrome	24	100%
Septic Joints	24	100%
Open Fractures	24	100%
Closed Fractures	24	100%
Nonunion/Malunion	4	17%
Tendon/Ligament Repair	17	71%
Irreducible Dislocations	23	96%
Other	4	17%
Other responses: Some “time dependent” nonunions in the midst of staged treatment; urgent and emergent cases as well as “time sensitive” fracture cases; Dysvascular limbs; Pelvis		

Nineteen (79%) respondents reported that operative indications or transfer policy for closed fractures have not changed. Five (21%) respondents reported that operative fracture or transfer indications at their institution have changed. These respondents noted the following changes: “Any fractures with good light reconstructive options or reasonable non-operative management/reduction being non-oped”; “Referring more malleolar ankle fractures to partners who can operate on them in surgery centers that are still open part time”; “Increase in outpatient fracture treatment at the ambulatory surgical center”; “It factors into non-op decision making for clavicles, distal fibula, and essentially all fractures where non-op is an option”.

COVID-19 Exposure and Infections Among Orthopaedic Trauma Team Members

The majority of respondents (15) reported that no one from their service has tested positive for COVID-19. Five program directors reported that ancillary staff have tested positive for COVID-19. One respondent noted that an attending staff had tested positive and another noted a resident had tested positive. 88% of respondents reported that they were satisfied with their institution’s precautions. Those who were unsatisfied with implemented precautions cited “slow preparation” and “lack of PPE” as their primary issues.

Future Directions

Respondents were also polled for future directions of COVID-19 precautions. Centers that have not implemented all of the precautions listed in our survey noted they plan to implement additional precautions in the near future. Other ideas for beneficial changes to the COVID-19 response included calls for “routine/universal COVID-19 testing,” “having non-trauma faculty cover trauma rooms,” and “sterilization of masks.”

## Discussion

This study is the first of its kind compiling and summarizing the early response of academic orthopaedic surgery division to the COVID-19 pandemic. In general, we found that orthopaedic trauma surgeons have been proactive in altering their team structures in order to mitigate the effects of the pandemic on their team members and their patients. In particular, we noted that many orthopaedic trauma teams successfully compartmentalized team members into self-contained units and transitioned to a virtual sign out early in the course of the pandemic. Additionally, at all responding centers, elective case volumes were drastically decreased in order to ensure that urgent and emergent care could continue for traumatized patients concurrent with preparations for a large influx of COVID-19 patients. Fortunately, many centers noted a decrease in trauma volumes likely due to the ongoing shelter in place orders active in many communities across the country.

In spite of these admirable efforts, some orthopaedic trauma divisions have had team members infected by the virus already, consistent with the role that orthopaedic trauma teams and orthopaedic teams in general play as frontline responders in patient care. Notably, many responding programs have not yet implemented standard PPE for all patient encounters or the routine use of N-95 masks in the operating room. The extent to which those changes would protect team members from infection is unclear, but the lack of standardization across programs speaks to a need for clearer guidance on the subject.

This information will hopefully be useful to orthopaedic surgeons attempting to decide on the best way to restructure their clinical teams in response to the continuing COVID-19 pandemic and any potential future pandemics. Additionally, it will hopefully serve as a baseline for future studies on the overall effectiveness of the early pandemic mitigation strategies undertaken by orthopaedic trauma teams.

Limitations of this study are primarily those associated with all survey studies. These include recall bias, although this was hopefully minimized by the recency of the events in question, and self-selection bias. In spite of these weaknesses, we feel that this information has value as an early guide for orthopaedic surgeons attempting to restructure their practices to respond effectively to the COVID-19 pandemic. Further studies will be needed to assess the effectiveness of the measures taken by orthopaedic trauma teams in the early phase of this pandemic, particularly with regard to protecting their team members and their patients from infection.

## Conclusions

Numerous orthopaedic trauma centers have been proactive in altering their team structures in order to mitigate the effects of the pandemic on their team members and patients. Successful compartmentalization of team members into self-contained units and transition to a virtual sign out early in the course of the pandemic was crucial to enabling the continued care of orthopaedic trauma patients. Additionally, elective case volumes were drastically decreased in order to ensure that urgent and emergent care could continue for traumatized patients concurrent with preparations for a large influx of COVID-19 patients. Fortunately, many centers noted a decrease in trauma volumes likely due to the ongoing shelter in place orders active in many communities across the country.
